# Small Molecules Targeting H3K9 Methylation Prevent Silencing of Reactivated *FMR1* Alleles in Fragile X Syndrome Patient Derived Cells

**DOI:** 10.3390/genes11040356

**Published:** 2020-03-27

**Authors:** Daman Kumari, Nicholas Sciascia, Karen Usdin

**Affiliations:** 1 Laboratory of Cell and Molecular Biology, National Institute of Diabetes, Digestive and Kidney Diseases, National Institutes of Health, 8 Center Drive, Bethesda, MD 20892, USA; nicholas.sciascia@gmail.com (N.S.); karenu@niddk.nih.gov (K.U.); 2 Laboratory of Genome Integrity, National Cancer Institute, Bethesda, MD 20892, USA

**Keywords:** fragile X syndrome, histone methyl-transferase, *FMR1* gene reactivation, DNA methylation, H3K9 methylation, chaetocin, DZNep, BIX01294

## Abstract

In fragile X syndrome (FXS), expansion of a CGG repeat tract in the 5′-untranslated region of the *FMR1* gene to >200 repeats causes transcriptional silencing by inducing heterochromatin formation. Understanding the mechanism of *FMR1* silencing is important as gene reactivation is a potential treatment approach for FXS. To date, only the DNA demethylating drug 5-azadeoxycytidine (AZA) has proved effective at gene reactivation; however, this drug is toxic. The repressive H3K9 methylation mark is enriched on the *FMR1* gene in FXS patient cells and is thus a potential druggable target. However, its contribution to the silencing process is unclear. Here, we studied the effect of small molecule inhibitors of H3K9 methylation on *FMR1* expression in FXS patient cells. Chaetocin showed a small effect on *FMR1* gene reactivation and a synergistic effect on *FMR1* mRNA levels when used in combination with AZA. Additionally, chaetocin, BIX01294 and 3-Deazaneplanocin A (DZNep) were able to significantly delay the re-silencing of AZA-reactivated *FMR1* alleles. These data are consistent with the idea that H3K9 methylation precedes DNA methylation and that removal of DNA methylation is necessary to see the optimal effect of histone methyl-transferase (HMT) inhibitors on *FMR1* gene expression. Nonetheless, our data also show that drugs targeting repressive H3K9 methylation marks are able to produce sustained reactivation of the *FMR1* gene after a single dose of AZA.

## 1. Introduction

Fragile X syndrome (FXS) is the leading cause of inherited intellectual disability, which affects approximately 1 in 5000 males and 1 in 4000 to 8000 females [1]. In addition to learning difficulties, behavioral problems including attention deficit, anxiety and autism spectrum disorder are frequent comorbid features [2]. FXS results from mutations in the fragile X mental retardation 1 (*FMR1*) gene that lead to a loss of functional fragile X mental retardation protein (FMRP). The most common mutation observed in FXS patients is the expansion of a CGG repeat tract in the 5’-untranslated region (UTR) of the *FMR1* gene to >200 repeats. Alleles with this repeat number are known as full mutation (FM) alleles. Such alleles are associated with epigenetic changes that lead to transcriptional gene silencing [3,4,5]. In addition to DNA methylation and hypoacetylated histones, the silenced *FMR1* gene in FXS patient cells is associated with the marks of facultative heterochromatin, histone H3 di-methylated at lysine 9 (H3K9me2) and histone H3 tri-methylated at lysine 27 (H3K27me3), as well as the marks of constitutive heterochromatin, histone H4 tri-methylated at lysine 20 (H4K20me3) and histone H3 tri-methylated at lysine 9 (H3K9me3) [6,7,8]. While H3K9me2 and H3K27me3 are distributed broadly on the *FMR1* locus, the constitutive heterochromatin marks peak near the CGG repeats, suggesting that the signal for their deposition is inherent in the expanded repeats [8]. The exact mechanism of repeat mediated heterochromatin formation in FXS is not known, but recent studies implicate *FMR1* mRNA in this process [9,10,11]. Given that the CGG expansion mutation is outside of the protein coding sequence, reactivation of the *FMR1* gene could be a potential treatment approach for FXS. However, a better understanding of the epigenetic silencing mechanism is needed to assess whether this is feasible and if so, to design an optimal reactivation strategy.

Inhibition of DNA methylation by treatment with 5-azadeoxycytidine (AZA) can partially restore *FMR1* gene expression in FXS patient cells in vitro. However, AZA treatment does not remove repressive histone methylation marks from the reactivated *FMR1* alleles [10]. This suggests that histone methylation marks are either deposited prior to DNA methylation or their deposition occurs independently of DNA methylation. We have previously shown that AZA treatment increases the levels of H3K27me3 on reactivated alleles [10]. While treatment with small molecules that inhibit Enhancer of zeste homolog 2 (EZH2) activity, and thus H3K27me3, does not reactivate the FM alleles, it does prevent re-silencing of reactivated alleles seen after AZA withdrawal [12]. This suggests that H3K27me3 occurs prior to DNA methylation and is important for gene silencing or that it may recruit other chromatin modifiers that are required for maintaining *FMR1* gene silencing. In contrast, the role of H3K9 methylation in *FMR1* gene silencing is not clear. In cells derived from FXS patients, the silenced *FMR1* gene is enriched for both H3K9me2 [7,8] and H3K9me3 marks [8]. In addition, the levels of SUV39H1, the histone methyl-transferase (HMT) responsible for H3K9me3, are reported to be increased on FM alleles in FXS patient cells [11]. Furthermore, some studies have shown that an increase in H3K9 methylation is associated with differentiation-induced gene silencing in FXS embryonic stem cells [9,13]. However, in lymphoblastoid cell lines derived from individuals carrying a transcriptionally active FM allele, H3K9me2 was present without DNA methylation [14,15]. This suggests that although this mark might be essential, it is not sufficient for *FMR1* gene silencing. Therefore, the precise role of H3K9 methylation in the *FMR1* silencing cascade remains unclear. 

In mammals, there are four major HMTs that methylate H3K9: G9a, GLP (G9a-like protein), SUV39H1/H2 and SETDB1 [16]. G9a and GLP are the main HMTs for mono- and di- methylation of H3K9 in euchromatin, while SUV39H1/H2 are important for H3K9me3 in pericentromeric heterochromatin [17]. Small molecule inhibitors for these HMTs have been described. Chaetocin is a fungal toxin that specifically inhibits the enzymatic activities of SUV39H1, G9a, GLP and ESET/SETDB1 [18]. Another inhibitor, BIX01294, was originally reported to be specific for G9a [19] but has also been shown to be active against GLP [20]. A more potent and selective inhibitor of G9a and GLP, UNC0638 has also been described [21]. Additionally, 3-Deazaneplanocin A (DZNep) [22], a known inhibitor of S-adenosylhomocysteine hydrolase, which inhibits H3K27me3 and H4K20me3 [23,24], was also shown to reduce the expression of SETDB1 and thus the levels of H3K9me3 [25]. 

To study the role of H3K9 methylation in *FMR1* gene silencing, we treated FXS patient cells with these inhibitors either alone or in combination with AZA, and monitored their effect on *FMR1* gene expression. Treatment with inhibitors of H3K9 methylation without first removing DNA methylation was not very effective, with only chaetocin showing some effect on *FMR1* reactivation in FXS patient cells. However, once the gene was reactivated by AZA, treatment with chaetocin showed a synergistic effect. In addition, BIX01294, chaetocin and DZNep delayed re-silencing of reactivated alleles in FXS lymphoblastoid cells. These results are consistent with previous studies that suggested that H3K9 methylation occurs prior to DNA methylation [13,14]. Moreover, our data provide further support that targeting both DNA methylation and histone methylation may be required for efficient reactivation of the *FMR1* gene. 

## 2. Materials and Methods 

### 2.1. Cell Lines 

All the cells used in this study were from male individuals and are listed in Table 1. FXS patient fibroblast (SC128 and SC126) and control fibroblasts (SC207) were a gift of Dr. Phil Schwartz (Children’s Hospital of Orange County Research Institute, California). The control fibroblasts BJ were obtained from Stemgent (San Diego, CA, USA). The FXS fibroblast cell line GM05848 was obtained from Coriell Cell Repositories (Coriell Institute for Medical Research, Camden, NJ, USA), and the FXS fibroblasts C10700 and C10259 were obtained from Carl Dobkin (Department of Human Genetics, New York State Institute of Basic Research in Developmental Disabilities, New York, NY, USA). All the fibroblasts cell lines used in this study have been described previously [26]. The control (GM06865) and FXS patient (GM04025, GM07294 and GM0032B) lymphoblastoid cell lines were obtained from Coriell Cell Repositories. Induced pluripotent stem cells (iPSCs) derived from SC128 fibroblasts were obtained from Phil Schwartz and were differentiated into neural stem cells (NSCs) by XCell Science (Novato, CA, USA) and have been described before [27]. Control BC1 NSCs were obtained from XCell Science and have also been described before [27]. The iPSCs lines, 13-1 and 15C, were derived from C10700 and C10259 fibroblasts, respectively, using an integration-free protocol [28] and differentiated into NSCs using the STEMdiff^TM^ Neural System (STEMCELL Technologies, Vancouver, British Columbia, Canada) as per manufacturer′s instructions. Briefly, single-cell suspension of iPSCs was prepared using StemPro® Accutase® (Thermo Fisher Scientific, Waltham, MA, USA), and 4 × 10^6^ cells were plated in 1 well of an 8-well AggreWell^TM^ 800 plate (STEMCELL Technologies, catalog number 27865) in 2 mL of STEMdiff^TM^ Neural Induction Medium (STEMCELL Technologies, catalog number 05831) supplemented with 10 µM ROCK inhibitor (Stemgent) and grown as embryoid bodies (EBs) for 5 days with daily partial (3/4) medium change. EBs were harvested as per manufacturer′s guidelines using a large 37 µm reversible strainer (STEMCELL Technologies, catalog number 27250) and plated on 1 well of Poly-L-Ornithine (Sigma-Aldrich, St. Louis, MO) and Laminin (Roche Applied Science, Germany) (PLO/L)-coated 6-well plate in 2 mL of STEMdiff^TM^ Neural Induction Medium. The EBs were grown further for 5 days and the medium was changed daily. Neural rosettes were collected using the STEMdiff Neural Rosette selection reagent (STEMCELL Technologies, catalog number 05832) and plated in 1 well of a 6-well plate coated with PLO/L in STEMdiff^TM^ Neural Induction Medium and grown with daily media change till ready to passage. The NSCs were collected using StemPro® Accutase® and plated on Geltrex^TM^- (Thermo Fisher Scientific) coated plates in Neural Expansion Medium (NEM) containing Neurobasal medium (Thermo Fisher Scientific), 1X GlutaMAX^TM^ (Thermo Fisher Scientific), 1X non-essential amino acids (NEAA, Thermo Fisher Scientific), 1X B27 supplement (Thermo Fisher Scientific) and 10–20 ng/µL β−FGF (R&D Biosystems, Minneapolis, MN). For neuronal differentiation, SC128 and BC1 NSCs were plated at 55,000 cells/well in 12-well plate in Neural Differentiation Medium (NDM) containing Neurobasal medium, 1X GlutaMAX^TM^, 1X NEAA, 1X B27 supplement and 1X N2 supplement (Thermo Fisher Scientific) and differentiated for two weeks before using them for drug treatments.

### 2.2. Drug Treatments

Lymphoblastoid cells were grown in Roswell Park Memorial Institute (RPMI) 1640 medium supplemented with 10% fetal bovine serum (FBS) and 1X antibiotic-antimycotic (all from Thermo Fisher Scientific). Fibroblasts were maintained in Dulbecco’s modified Eagle’s medium (DMEM) supplemented with 10% FBS, 1X NEAA and 1X antibiotic–antimycotic. Fibroblasts were grown until 75–85% confluent in 12-well tissue culture plates before adding drugs. Approximately 120,000 NSCs were plated in NEM on 24-well plates coated with Geltrex^TM^ and allowed to become 70% confluent before drug treatments. NSCs were differentiated into neurons for two weeks before treating with compounds. The lymphoblastoid cells, fibroblasts, NSCs and neurons were treated with HMT inhibitors for 48 h. For combination drug treatments, cells were treated with AZA for 72 h followed by 48 h treatment with HMT inhibitors. Drug concentrations used are mentioned in the figure legend.

### 2.3. RNA Isolation and Quantitative Reverse Transcriptase Polymerase Chain Reaction (RT-qPCR) 

Total RNA was isolated from untreated or treated cells using TRIzol^TM^ reagent (Thermo Fisher Scientific) and quantified on NanoDrop ND-1000 (Thermo Fisher Scientific). Three hundred nanograms of total RNA was reverse transcribed in 20 µL final volume using SuperScript®VILO^TM^ master mix (Thermo Fisher Scientific) as per manufacturer’s instructions. Real-time PCR was performed in triplicate using 2 µL of the cDNA, FAM-labeled *FMR1* and VIC-labeled glyceraldehyde 3-phosphate dehydrogenase (*GAPDH*) or β-glucoronidase *(GUS)* probe-primers (Thermo Fisher Scientific) and TaqMan^®^ FAST universal PCR master mix (Thermo Fisher Scientific) on StepOnePlus^TM^ Real-Time PCR system (Thermo Fisher Scientific). 

### 2.4. DNA Isolation and PCR

Genomic DNA was isolated from treated cells using the salting-out method [29]. The number of CGG repeats in the *FMR1* gene and DNA methylation levels were analyzed by PCR and Southern blotting as described earlier [26,30]. In DZNep-treated samples, DNA methylation at 22 CpG residues in the *FMR1* promoter was analyzed by pyrosequencing of bisulfite-converted DNA by EpigenDx, Inc. (Hopkinton, MA, USA) using assays ADS1451-FS1 and ADS1451-FS2 as described previously [31].

### 2.5. Chromatin Immunoprecipitation (ChIP)

ChIP assays were performed as described before [10] using a ChIP assay kit from EMD-Millipore (Billerica, MA, USA). To prepare chromatin for immunoprecipitation, cells were fixed with 1% formaldehyde for 10 min at room temperature and lysed as per the kit manufacturer’s instructions. The chromatin was sonicated into <500 bp fragments using a Bioruptor® (Diagenode, Denville, NJ, USA) for 6 min with 30 sec ON and 30 s OFF at medium setting. Twenty micrograms of chromatin were used in each immunoprecipitation reaction. The following antibodies (5 µg/reaction) were used for ChIP: H3K9me2 (Abcam, ab1220), H3K9me3 (Abcam, ab8898), H3K27me3 (EMD-Millipore, 07-449), H3K4me2 (Abcam, ab7766), normal mouse IgG (EMD-Millipore, 12-371) and normal rabbit IgG (EMD-Millipore, 12-370). Real-time PCRs on the immunoprecipitated DNAs were carried out in triplicate in 20 µL final volume using the Power SYBR™ Green PCR master mix (ThermoFisher Scientific) and 200 nM of each primer and 2 µL of DNA. For amplification of *FMR1* exon1, the primer pair Exon 1-F (5′-CGCTAGCAGGGCTGAAGAGAA-3′) and Exon1-R (5′-GTACCTTGTAGAAAGCGCCATTGGAG-3′) was used. This primer pair amplifies the region +236 to +312 relative to the transcription start site. For amplification of *FMR1* promoter region, primer pair *FMR1*-F (5′-GAACAGCGTTGATCACGTGAC-3′) and *FMR1*-R (5′-GTGAAACCGAAACGGAGCTGA-3′) was used. For the amplification of a control active genomic locus, *GAPDH* (accession number BC025925), primers hsGAPDH exon1F1 (5’-TCGACAGTCAGCCGCATCT-3’) and hsGAPDH intron1R1 (5’-CTAGCCTCCCGGGTTTCTCT-3’) were used. To amplify a control inactive locus, the *Sat2* repeat (accession number X72623), primers hsSat2 repeat F1 (5’-ATCGAATGGAAATGAAAGGAGTCA-3’) and hsSat2 repeat R1 (5’-GACCATTGGATGATTGCAGTCA-3’) were used. For quantitation, the comparative threshold (Ct) method was used. Enrichment in ChIP samples was calculated over 100% of input and normalized to AZA/DMSO treated samples. 

### 2.6. Western Blot Analyses

For making total cell lysates, cells were scraped in the growth medium and pelleted at 250 RCF for 6 min. The cell pellet was washed with ice-cold PBS supplemented with 1X protease inhibitor cocktail (Sigma-Aldrich) and 1X phosphatase inhibitor (Sigma-Aldrich). The pellet was then resuspended in the lysis buffer (10 mM Tris Cl pH 7.5, 1 mM EDTA pH 8.0, 1% Triton X-100, 1X protease inhibitor cocktail and 1X phosphatase inhibitor) and incubated on ice for 10 min followed by sonication for 30 s at medium setting using Bioruptor® to solubilize proteins and shear the DNA. The amount of protein in the lysate preparation was quantified using Bio-Rad Protein Assay Dye Reagent Concentrate (Bio-Rad Laboratories, Inc., Hercules, CA, USA) as per manufacturer’s protocol. Before using the lysate for Western blot analyses, equal volumes of 2X Novex® Tris-Glycine SDS Sample Buffer (Thermo Fisher Scientific) and 1X of NuPAGE® Sample Reducing Agent (Thermo Fisher Scientific) were added, and the sample was heated at 95 °C for 10 min.

For the detection of histone proteins, 10 µg of total cell lysates were run on a 4–20% NuPage Tris-Glycine polyacrylamide gel (Thermo Fisher Scientific) for 80–90 min at 125 V and transferred to nitrocellulose membrane (0.2 µm pore size, Thermo Fisher Scientific) using Trans-Blot® Turbo^TM^ (Bio-Rad Laboratories). The membrane was blocked for one hour with 5% blocking agent (GE Healthcare Bio-Sciences, Pittsburg, PA) in TBST (1X Tris buffered saline with 0.1% Tween 20). The blot was incubated with 1:5000 dilution of the following primary antibodies from Abcam (Cambridge, MA, USA) overnight at 4 °C: H3K9me2 (ab1220), H3K9me3 (ab8898), Total H3 (ab1791), and then probed with 1:5000 diluted HRP-labeled secondary antibody (GE Healthcare Bio-Sciences). The signal was detected using ECL^TM^ Prime detection reagents (GE Healthcare Bio-Sciences) and imaged with FluorChem^TM^ M (Proteinsimple, Santa Clara, CA, USA) and quantified using AlphaView software (Proteinsimple). The blot was then stripped with Restore^TM^ Western Blot stripping buffer (Thermo Fisher Scientific) and re-probed with 1:5000 dilution of anti-histone H3 (Abcam, ab1791). The amount of methylated histones was normalized to the amount of total H3.

### 2.7. Immunostaining

Immunostaining for stem cell marker SSEA-4 was done using BD Pharmingen^TM^ Alexa Fluor® 488 mouse anti-SSEA-4 (BD, Franklin Lakes, NJ, #560308, 1:100) for 1 hour at room temperature as per manufacturer’s instructions. Immunostaining for all other stem cell and neuronal markers was performed as described earlier [31]. The following unlabeled antibodies were used: Oct4 (Stemgent, #09-0023, 1:100), Nanog (EMD Millipore, #MABD24, 1:500), SOX2 (EMD-Millipore, #AB5603, 1:500), Nestin (EMD Millipore, #AB5922, 1:1000), PAX 6 (Covance, #PRB-278P, 1:200) and MAP2 (Sigma-Aldrich, #M2320, 1:500). After the incubation with the primary antibodies overnight, the cells were incubated with 1:500 diluted secondary antibody labeled with either Alexa-Fluor®488 or Alexa-Fluor® 555 (Thermo Fisher Scientific) at room temperature for 45 min. The nuclei were counterstained with DAPI for 15 min at room temperature and the images acquired using EVOS®FL Microscope (Thermo Fisher Scientific).

### 2.8. Statistical Analysis

Statistical significance of observed differences between cells treated with the vehicle or indicated small molecule inhibitor was calculated using paired two-tailed Student’s *t* test (GraphPad Software, Inc., La Jolla, CA, USA), and a *p*-value of ≤0.05 was considered statistically significant in this exploratory study. Other statistical details can be found in the figure legends. 

## 3. Results

### 3.1. Small Molecules Targeting H3K9 Methylation, by Themselves, are not Very Effective at Activating FMR1 Expression in FXS-Patient-Derived Cells

**Figure 1:**
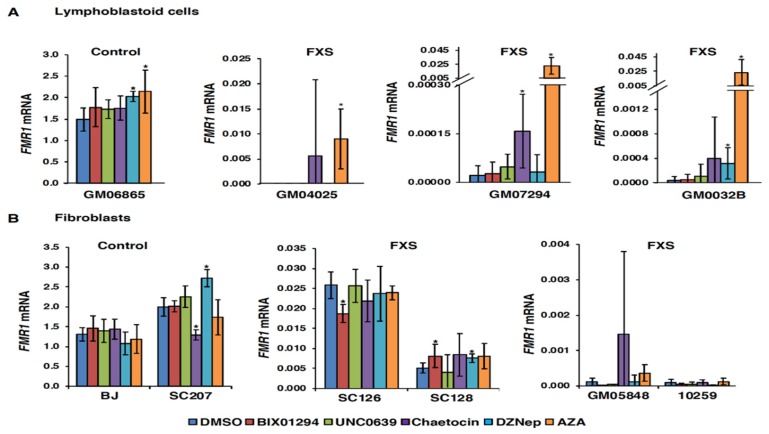
Effect of histone methyl-transferase (HMT) inhibitors on *FMR1* gene expression in lymphoblastoid (**A**) and fibroblast (**B**) cell lines. Control and fragile X syndrome (FXS) cells were treated with DMSO, 4 µM of BIX01294, 100 nM chaetocin, 0.5 µM UNC0638 or 5 µM DZNep for 48 h and 10 µM 5-azadeoxycytidine (AZA) for 72 h (lymphoblastoid) or 1 µM AZA for 72 h (fibroblasts). Cells were harvested at the end of the treatment for total RNA. The levels of *FMR1* mRNA were analyzed by RT-qPCR and are shown as a percentage of *GAPDH* mRNA. Data shown are an average of at least three independent treatments, and error bars represent standard deviation. Statistically significant differences are indicated by an asterisk (*p* < 0.05).

Previous studies have shown that treatment with the DNA methylation inhibitor AZA can partially reactivate the *FMR1* gene in FXS-patient-derived lymphoblastoid and fibroblast cell lines [7,10,32,33]. The reactivated alleles show reduced DNA methylation and an increase in the histone acetylation at the *FMR1* locus [7,34]. However, AZA treatment does not reduce the level of repressive H3K9 methylation on the *FMR1* gene [10]. To study the relative contribution of the H3K9 methylation to *FMR1* gene silencing, we treated FXS cells with known inhibitors of H3K9 methylation (chaetocin, BIX01294 and UNC0638) and a global histone methylation inhibitor (DZNep) and analyzed the levels of *FMR1* mRNA. We chose the concentrations based on the previous studies [18,19,21,24,25,35,36,37,38,39,40,41] and also based on the observed toxicity in the different cell types used in this study. We found that only chaetocin was able to reactivate the *FMR1* gene in every FXS lymphoblastoid cell line tested in this study (Figure 1A). However, the effect was variable between cell lines and much lower than the reactivation level achieved with AZA treatment (Figure 1A). Similar small or inconsistent effects on the *FMR1* mRNA levels were seen with the HMT inhibitors when used alone in fibroblasts (Figure 1B). These observations suggest that, when used alone, these HMT inhibitors are not effective at reactivation of the *FMR1* gene. This would be consistent with the dominant role of DNA methylation in *FMR1* gene silencing [12,42]. 

**Figure 2:**
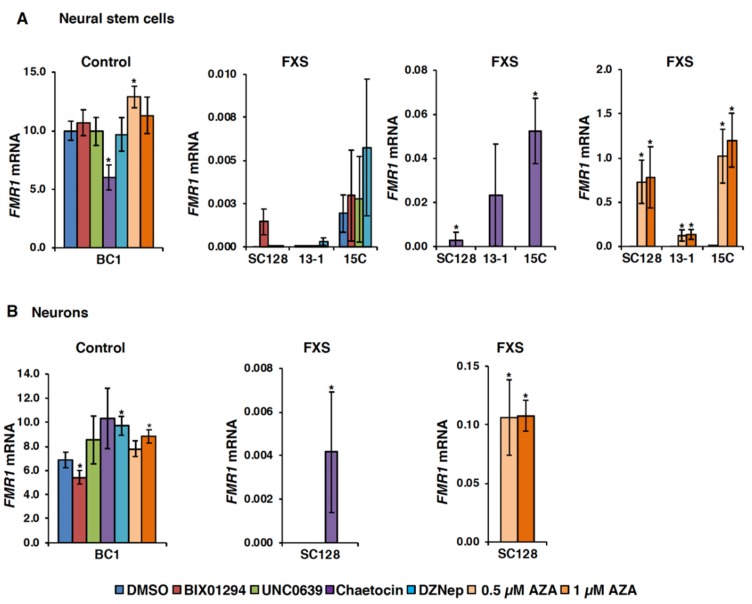
Effect of AZA and HMT inhibitors on the *FMR1* mRNA levels in neural stem cells (**A**) and neurons (**B**). Cells were treated with DMSO or HMT inhibitors, 4 µM BIX01294, 100 nM chaetocin, 0.5 µM UNC0638 or 5 µM DZNep for 48 h and with 0.5 µM or 1 µM AZA for 72 h. *FMR1* mRNA levels were analyzed by RT-qPCR and are shown as a percentage of *GAPDH* mRNA. The error bars indicate standard deviation from at least four independent treatments. Statistically significant differences are indicated by an asterisk (*p* < 0.05).

To study the effect of these inhibitors on *FMR1* expression in disease-relevant neural cells, we generated induced pluripotent stem cells (iPSCs) from three different FXS fibroblast cell lines and then differentiated these into neural stem cells (NSCs) and neurons as described in the Materials and Methods section (Figure S1 for 13-1, Figure S2 for 15C). AZA was used at a lower concentration in NSCs and neurons because of its toxicity in these cells. Nonetheless, reactivation of the *FMR1* gene was observed in all the three FXS NSC lines even with a lower dose (0.5 µM-1 µM) of AZA (Figure 2A, far right panel). Treatment with the HMT inhibitors alone did not significantly affect *FMR1* mRNA levels in control NSCs except for chaetocin, which significantly decreased *FMR1* mRNA levels (Figure 2A, far left panel). BIX01294 and chaetocin showed a low level of *FMR1* reactivation in FXS NSCs. However, the effect was variable and much lower compared to AZA. It should be noted that a very low level of *FMR1* mRNA was detected in one FXS NSC line (15C) after DMSO treatment, and some increase over the basal level was observed with DZNep, but it did not reach statistical significance. Thus, as with fibroblasts and lymphoblastoid cells, these compounds were also not effective in NSCs when used alone (Figure 2A). Similarly, AZA reactivated FM alleles in neurons (Figure 2B, right panel), but of the HMT inhibitors, only chaetocin treatment resulted in any detectable expression of *FMR1* mRNA (Figure 2B, middle panel). 

It should be noted that while the parent fibroblast SC128 cell line is mosaic for the presence of premutation (PM) and FM alleles and a low level of *FMR1* mRNA can be detected in untreated or vehicle (DMSO) treated cells, both the NSCs and neurons were derived from an iPSC clone that carries a single methylated allele and does not express the *FMR1* gene at all [27]. Thus, the effect of chaetocin in the neural cell lines reflects reactivation of the silenced FM alleles and not increased expression from an active PM allele.

### 3.2. Chaetocin Potentiates the Expression of AZA-Reactivated FM Alleles in FXS Patient Cells

**Figure 3:**
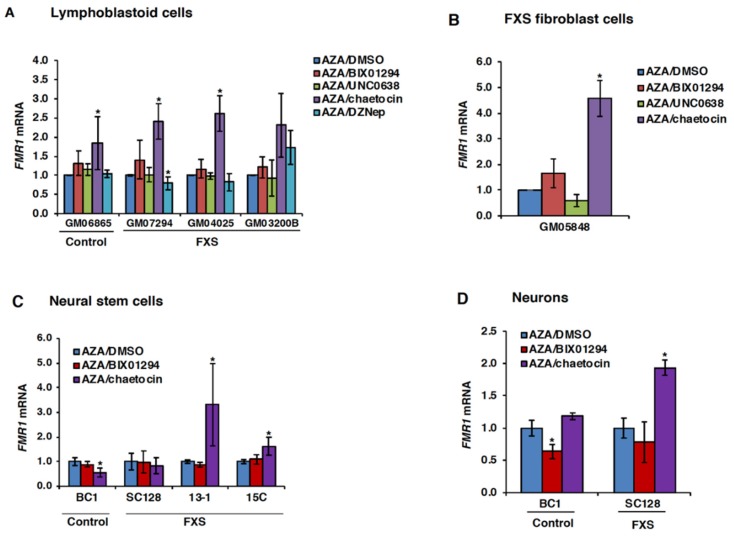
Effect of HMT inhibitors on *FMR1* gene expression after pretreatment with AZA in human lymphoblastoid cell lines (**A**), FXS fibroblast cell line (**B**), neural stem cells (**C**) and neurons (**D**). Lymphoblastoid and fibroblast cells were treated with 10 µM AZA for 72 h followed by either DMSO or indicated HMT inhibitors (4 µM of BIX01294, 100 nM chaetocin, 0.5 µM UNC0638 or 5 µM DZNep) for the next 48 h and harvested for total RNA on day 5. The neural stem cells and neurons were treated with 0.5 µM AZA for 72 h and recovered in drug-free medium for 24 h, followed by treatment with indicated inhibitors for 48 h and harvested for total RNA on day 6. The levels of *FMR1* mRNA and *GAPDH* mRNA were analyzed by RT-qPCR. The *FMR1* mRNA levels were first normalized to *GAPDH* mRNA levels and are shown relative to the levels in AZA/DMSO at day 5 (**A**,**B**) or day 6 (**C**,**D**). Data shown are an average of at least three independent treatments, and error bars represent standard deviation. Statistically significant differences are indicated by an asterisk (*p* < 0.05).

The synergistic effect of AZA and various histone deacetylase inhibitors on *FMR1* gene expression has been reported [43]. To determine if there was any similar synergistic effect between AZA and inhibitors of H3K9 methylation, we treated FXS cells with AZA to initially reactivate the *FMR1* gene, followed by treatment with either DMSO or small molecule inhibitors of H3K9 methylation. When used in combination with AZA, chaetocin showed a synergistic effect in the control and all three FXS lymphoblastoid cell lines (Figure 3A), as well as in one FXS fibroblast cell line (Figure 3B). This result was statistically significant in three out of four FXS cell lines. The other HMT inhibitors tested did not show any significant effect on the *FMR1* mRNA levels in either lymphoblastoid or fibroblast cell lines when used in combination with AZA (Figure 3A,B). 

Next, we tested the effect of combined treatment with AZA and HMT inhibitors in FXS NSCs and neurons. Once again, chaetocin was the only HMT inhibitor to have any synergistic effect with AZA, significantly increasing *FMR1* mRNA levels in two out of three FXS NSC lines tested (Figure 3C), as well as in FXS neurons (Figure 3D). Thus, only chaetocin was able to potentiate the activity of AZA-reactivated FM alleles in all of the cell lines tested.

### 3.3. HMT Inhibitors Delay Re-Silencing of AZA-Reactivated Alleles in FXS Lymphoblastoid Cells

**Figure 4:**
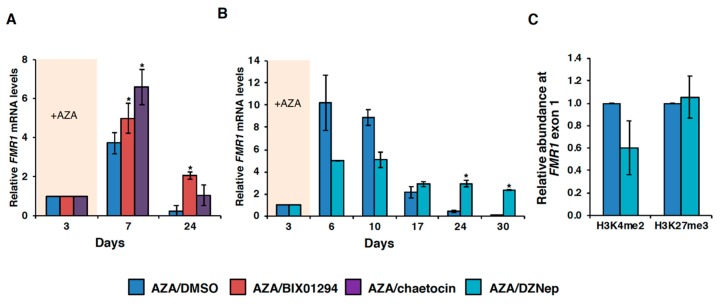
(**A**,**B**) Effect of BIX01294, chaetocin and DZNep in delaying re-silencing of AZA reactivated FM allele in FXS lymphoblastoid cell line GM04025. Cells were treated with 10 µM AZA for 72 h and split and treated with either DMSO or indicated HMT inhibitor for up to 30 d, adding fresh media and HMT inhibitor every 3 d. The *FMR1* mRNA levels were measured by RT-qPCR and normalized to the levels of *GUS* mRNA and are shown relative to day 3. Data shown are from at least two independent treatments. (**C**) The levels of histones H3K4me2 and H3K27me3 on *FMR1* exon1 were analyzed in GM04025 cells treated with AZA/DMSO or AZA/DZNep at day 10 by ChIP-qPCR. Data are shown relative to AZA/DMSO and are an average of three independent experiments. Error bars represent standard deviation, and statistically significant differences are indicated by an asterisk (*p* < 0.05).

AZA-mediated *FMR1* gene reactivation is transient, such that after removal of AZA from the culture media, the gene is re-silenced in ~21–24 d [7,10]. Given the toxicity of AZA, the identification of compounds able to maintain *FMR1* expression after a single dose of AZA may be therapeutically useful. Because H3K9 methylation is not erased by AZA treatment [10], we hypothesized that treatment of cells with compounds capable of removing this mark might prevent re-silencing of the *FMR1* gene after AZA withdrawal. To test this, we treated FXS lymphoblastoid cells with 10 µM AZA for 72 h, followed by either DMSO, BIX01294, chaetocin or DZNep for 24–30 d. Treatment with all three of these HMT inhibitors significantly delayed the re-silencing of AZA-reactivated FM alleles in FXS cells (Figure 4A-B, Figure S3). In particular, in cells treated with these compounds, the FM allele was as active or more active after 24 or 30 d of treatment than they were after three days of AZA treatment and 4–100 times more active than the cells treated with AZA followed by DMSO.

Given that DZNep affects H3K27 trimethylation [23] and a decrease in H3K27me3 prevents re-silencing of reactivated alleles [12], we analyzed H3K27me3 levels in AZA/DMSO and AZA/DZNep treated GM04025 cells at day 10. There was no decrease in the levels of H3K27me3 after DZNep treatment (Figure 4C), suggesting that the effect of DZNep on gene re-silencing was not mediated via an effect on H3K27me3. Additionally, there was no significant change in the DNA methylation levels after DZNep treatment (Figure S4). However, there was a slight decrease in the levels of the positive histone methylation mark H3K4me2 on the *FMR1* gene after DZNep treatment (Figure 4C). Because H4Kme2 is associated with increased transcription, it might explain the reduced levels of *FMR1* mRNA levels after DZNep treatment at day 10. 

### 3.4. Effect of HMT Inhibitors on H3K9 Methylation of the FMR1 Gene in FXS Patient Cells

**Figure 5:**
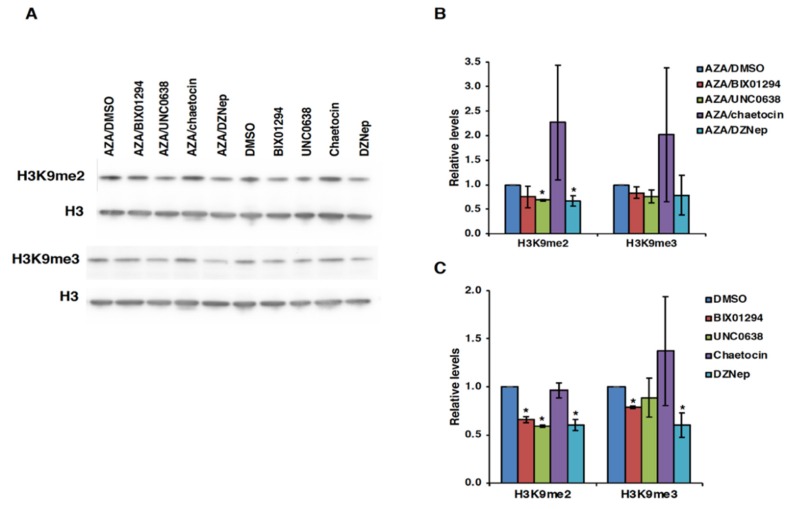
Western blot analyses for H3K9me2 and H3K9me3 in FXS cell line GM04025. (**A**) A representative western blot is shown. (**B**,**C**) Quantitation from three independent western blot experiments. Cells were treated with either HMT inhibitors alone or in combination with AZA as described in the legend for Figure 1 and Figure 3, respectively. Total cell lysates were prepared after 48 h treatment with HMT inhibitors alone or at day 5 after AZA/HMT inhibitor treatment. Total H3 levels were used for normalization. Error bars represent standard deviation, and statistically significant differences are indicated by an asterisk (*p* < 0.05).

Next, we wanted to determine if the observed effect of HMT inhibitors on *FMR1* mRNA levels was mediated via changes in H3K9 methylation levels. For this, we analyzed global changes in the levels of H3K9me2 and H3K9me3 by western blot analysis and on the *FMR1* gene specifically by ChIP assay. In GM04025 lymphoblastoid cells, a decrease in the levels of H3K9me2 was observed by western blot analysis after treatment with all HMT inhibitors except for chaetocin (Figure 5). 

**Figure 6:**
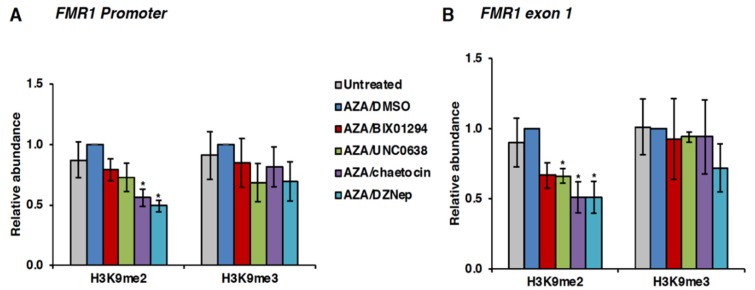
(**A**,**B**) Effect of HMT inhibitors on the levels of H3K9me2 and H3K9me3 on the *FMR1* promoter (**A**) and exon 1 (**B**) in the FXS cell line GM04025. Cells were treated with 10 µM AZA for 72 h followed by indicated HMT inhibitors as described in the legend for Figure 3. Chromatin was prepared at day 5 from treated and untreated cells and used in ChIP assay. Abundance of H3K9me2 and H3K9me3 in AZA/HMT inhibitor treated cells is shown relative to AZA/DMSO treated cells. Data shown are an average of three independent experiments, and error bars represent standard error of the mean (SEM). Statistically significant differences are indicated by an asterisk (*p* < 0.05).

However, in ChIP assays, chaetocin was also able to decrease the levels of H3K9me2 at the *FMR1* promoter and exon 1 (Figure 6). Interestingly, DZNep treatment decreased the levels of H3K9me2 both globally (Figure 5 and Figure S5) and on the *FMR1* gene (Figure 6). Thus, our data suggest that the decrease in the H3K9me2 levels on the *FMR1* gene in AZA/chaetocin- and AZA/DZNep-treated FXS cells may be responsible for their effect on delaying re-silencing after AZA withdrawal. A similar decrease in H3K9me2 was seen with BIX01294, although it did not reach statistical significance (Figure 6). It is possible that long-term treatment with BIX01294 can lower the levels of H3K9 methylation, which may be the reason for its effect on delaying re-silencing of reactivated FM alleles. Small decreases in H3K9me2 levels were also seen at an active control locus *GAPDH* and an inactive control locus, the *Sat2* repeats. However, these decreases only reached statistical significance for DZNep on the *Sat2* repeats (Figure S6). This suggests that although global decreases in the levels of H3K9me2 are seen after treatment with HMT inhibitors, these inhibitors do not affect at all genomic loci equally. This is consistent with the demonstration that other epigenetic modifying compounds, including AZA, only affect a limited number of genes [44,45,46].

## 4. Discussion and Conclusions 

In this study, we examined the effect of small molecule inhibitors of the HMTs responsible for H3K9 dimethylation and trimethylation on *FMR1* gene expression in different patient-derived cell types. Chaetocin was the only compound that was able to reactivate the silenced *FMR1* gene in all the cell types studied. However, the observed effect was quite small. One reason for this could be that DNA methylation locks in the silenced state and its removal may be necessary to activate transcription [42]. Consistent with this idea, chaetocin was more effective after AZA pretreatment in all cell types (Figure 3). A modest increase in the *FMR1* mRNA levels was also seen in AZA/BIX01294-treated FXS lymphoblastoid and fibroblast cells. 

While their effects on *FMR1* expression were quite limited, both chaetocin and BIX01294 were effective at delaying the re-silencing of AZA reactivated FM alleles in FXS cells (Figure 4A), with cells treated with these compounds showing 4–8 times more *FMR1* mRNA after 24 d in culture than the cells treated with vehicle alone and 1–2 times the level of *FMR1* mRNA seen after three days of AZA treatment. Interestingly, the global HMT inhibitor, DZNep, did not reactivate the *FMR1* gene in FXS cells and had no synergistic effect with AZA, but it was also able to prevent the re-silencing in lymphoblastoid cells, showing levels of *FMR1* mRNA after 30 d of treatment that were 100 times higher than vehicle-only cells and two times more than those observed after three days of AZA treatment. This is consistent with our previous observation that inhibition of the H3K27 methylase, EZH2, was not able to reactivate the *FMR1* gene in FXS cells, but once the DNA methylation was removed, it prevented re-silencing [12]. The present study provides further support that removal of DNA methylation is necessary to see the optimal effect of HMT inhibitors on *FMR1* expression in FXS cells.

Although DZNep is a broad-spectrum methyl-transferase inhibitor and has been shown to inhibit the activity of various HMTs including EZH2 [23,24], our data suggest that DZNep’s effect on preventing re-silencing was not mediated via reducing H3K27 methylation or DNA methylation but might be related to a decrease in H3K9me2 levels. Because DZNep, chaetocin and BIX01295 reduced the levels of H3K9me2 on the *FMR1* gene, this may explain their effect on the expression of AZA-reactivated alleles in FXS cells. Our inability to see gene reactivation with DZNep treatment alone differs from a previously published report [46]. We speculate this difference is due to either the higher concentration of DZNep (25 µM) they used, or the longer treatment time used in their study. 

The observed effect of inhibitors of H3K9 methylation on the sustained expression of the AZA-reactivated FM alleles in the present study is much lower than the effect we previously reported for EZH2 inhibitors that reduce H3K27me3. One reason for this could be the difference in the enrichment levels of H3K9 methylation and H3K27me3 on FM alleles [8]. In addition, there are multiple HMTs that modulate H3K9 methylation, and it is possible that the inhibition of one of them could be compensated by the activity of others. If so, the simultaneous targeting of a number of these proteins may have a larger effect on *FMR1* expression. Because DZNep and BIX01294 showed less toxicity compared to chaetocin, they may be more useful for combinatorial treatment with AZA for sustained expression of the *FMR1* gene.

Thus, our data provide a proof of concept for the idea that targeting epigenetic modifications like H3K9 methylation in combination with DNA demethylation may ultimately have therapeutic potential for FXS patients. Encouragingly, a recent study showed that ~35% of normal FMRP levels could be clinically beneficial [47], which suggests that high levels of reactivation may not be necessary. However, there are a number of challenges that need to be overcome before any gene reactivation approach could be clinically useful. Firstly, more effective and less toxic small molecule inhibitors of HMTs and other chromatin-modifiers are needed, and a better understanding of the mechanism of silencing would also be valuable in assessing whether a more *FMR1*-specific reactivation approach is possible. Secondly, since long CGG repeat tracts in mRNA inhibit translation [48,49], a combination of drugs that improve FMRP production with those that reverse gene silencing will also be needed. Finally, restoring expression from PM or FM alleles in FXS patients presents additional challenges due to the toxicity associated with the expression of CGG RNA (rCGG) [50,51,52]. A recent study showed that non-cleaving antisense oligonucleotides (ASOs) that selectively block CGG repeat-associated non-AUG (CGG RAN) translation also enhanced the production of FMRP [53]. Thus, combining small molecules that reactivate FM alleles with such ASOs might provide a good strategy to restore FMRP expression while reducing the negative consequences of rCGG toxicity. Since BIX01294 has also been shown to reduce CGG RAN translation [54], related compounds with similar properties may provide added benefit. Until recently, the lack of a suitable animal model for the gene silencing seen in human FM carriers has limited preclinical evaluation of the efficacy and toxicity of *FMR1*-reactivating drugs. However, the recent description of humanized mice containing brain implants of FXS iPSC-derived neurons should facilitate future preclinical testing of compounds optimized to deal with all of these issues [46].

In summary, our data provide further evidence for the idea that histone methylation marks are deposited on FM alleles prior to DNA methylation, and that DNA methylation plays a dominant role in maintaining *FMR1* gene silencing in FXS. Our data also suggest that a combination of drugs targeting DNA methylation and repressive histone methylation marks enriched on the *FMR1* gene will be necessary for sustained reactivation of the *FMR1* gene in FXS patient cells.

## Figures and Tables

**Table 1:** List of cell lines used in this study.

Cell Line Name	Cell Type	FXS/Control	CGG-Repeat Size/MethyLation
BJ	fibroblast	Control	26
SC207	fibroblast	Control	20
SC126	fibroblast	FXS	Mosaic for PM/ methylated FM
SC128	fibroblast	FXS	Mosaic for PM/ methylated FM
C10259	fibroblast	FXS	Methylated FM (1363 by SB)
C10700	fibroblast	FXS	Mosaic for PM/ methylated FM
GM05848	fibroblast	FXS	Methylated FM (~700)
GM06865	lymphoblastoid	Control	30
GM0032B	lymphoblastoid	FXS	Methylated FM (570)
GM04025	lymphoblastoid	FXS	Methylated FM (645)
GM07294	lymphoblastoid	FXS	Methylated FM (745)
13-1	iPSCs	FXS	Methylated FM (463)
15C	iPSCs	FXS	Methylated FM (330)
SC128	iPSCs	FXS	Methylated FM (300)
BC1 NSC	NSC	Control	NA
SC128 NSC	NSC	FXS	Methylated FM (300)
SC128 neurons	neurons	FXS	Methylated FM (300)

SB, Southern blot; PM, premutation; FM, full mutation; iPSCs, induced pluripotent stem cells; NSCs, neural stem cells; NA, not available.

## Supplementary Materials

The following are available online at https://www.mdpi.com/2073-4425/11/4/356/s1: Figure S1: Characterization of FXS iPSCs, 13-1 and its differentiation into neural stem cells; Figure S2: Characterization of FXS iPSCs, 15C and its differentiation into neural stem cells; Figure S3: Effect of DZNep treatment in delaying re-silencing in FXS cell line GM0032B; Figure S4: Effect of DZNep treatment on DNA methylation levels in FXS cell line GM04025; Figure S5: Western blot analysis for H3K9me2 and H3K9me3 in FXS cell line GM0032B; Figure S6: Effect of HMT inhibitors on the levels of H3K9me2 and H3K9me3 on *GAPDH* and *Sat 2* repeats in the FXS cell line GM04025.
