# Supplementary material for: Small Molecules Targeting H3K9 Methylation Prevent Silencing of Reactivated FMR1 Alleles in Fragile X Syndrome Patient Derived Cells
Source: Genes (Basel). 2020 Mar 27;11(4):356. doi: 10.3390/genes11040356 (PMC7230530; doi:10.3390/genes11040356)
Supplement: Supplementary file 1 [file genes-11-00356-s001.pdf]

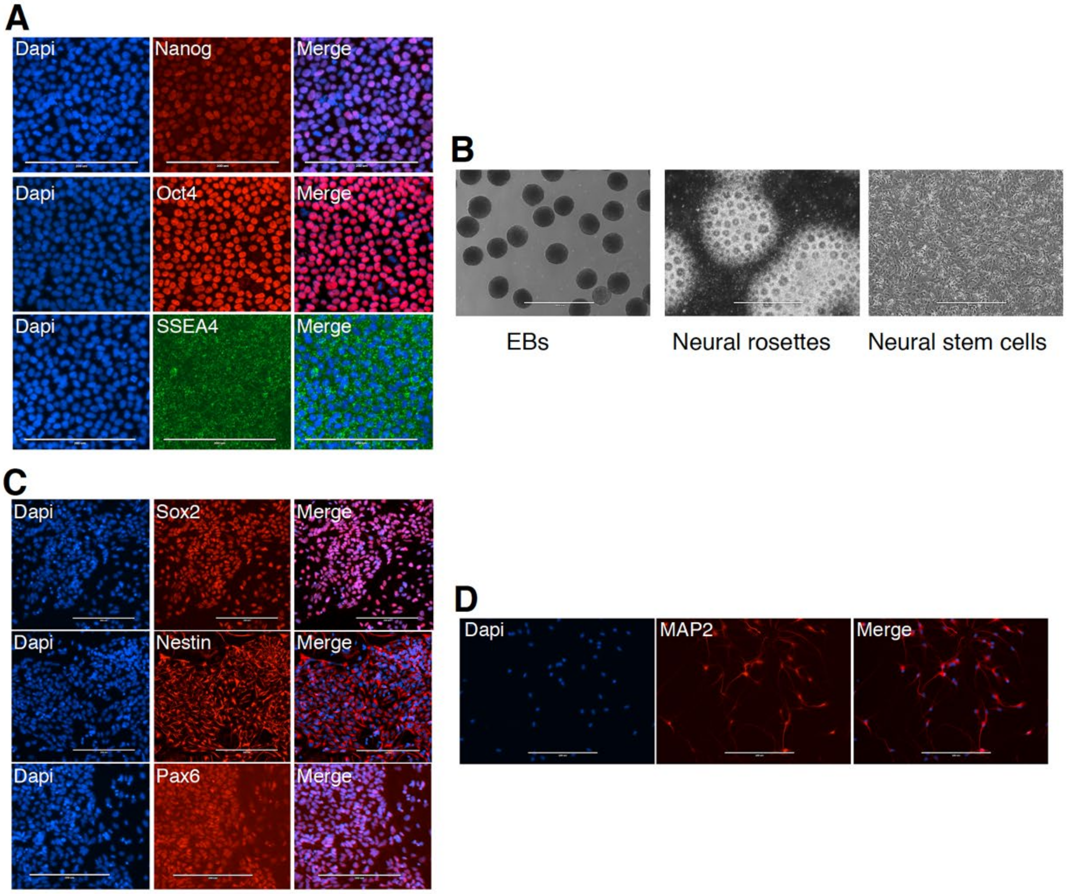

Figure S1. (A). Pluripotency marker staining for 13-1 iPSCs, Scale bar 200 μm. **(B)** Generation of neural stem cells (NSCs) from 13-1 iPSCs using AggreWell™ method. EBs, embryoid bodies, scale bar 400 μm for EBs and neural stem cells panels and 1000 μm for the panel showing neural rossettes. **(C)** NSC marker staining for 13-1 NSCs, scale bar 200 μm. **(D)** The 13-1 NSCs were differentiated into neurons that stain positive for the mature neuronal marker MAP2 at two weeks, scale bar 200 μm.

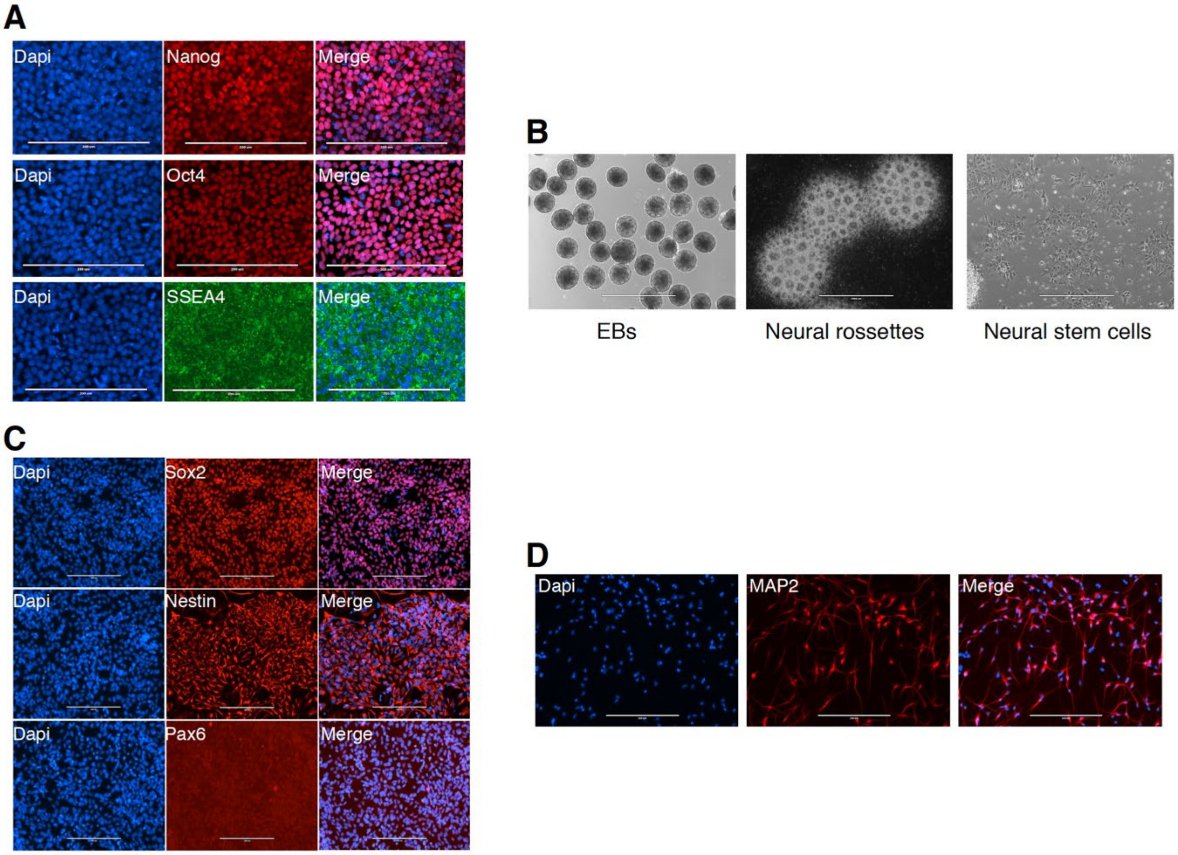

Figure S2. (A). Pluripotency marker staining for 15C iPSCs, Scale bar 200 µm. **(B)** Generation of neural stem cells (NSCs) from 15C iPSCs using AggreWell™ method. EBs, embryoid bodies at day 5, scale bar 1000 µm for EBs and neural rossettes (at day 5) panels and 400 µm for neural stem cells panel. **(C)** NSC marker staining for 15C NSCs, scale bar 200 µm. **(D)** The 15C NSCs were differentiated into neurons that stain positive for the mature neuronal marker MAP2 at two weeks, scale bar 200 µm.

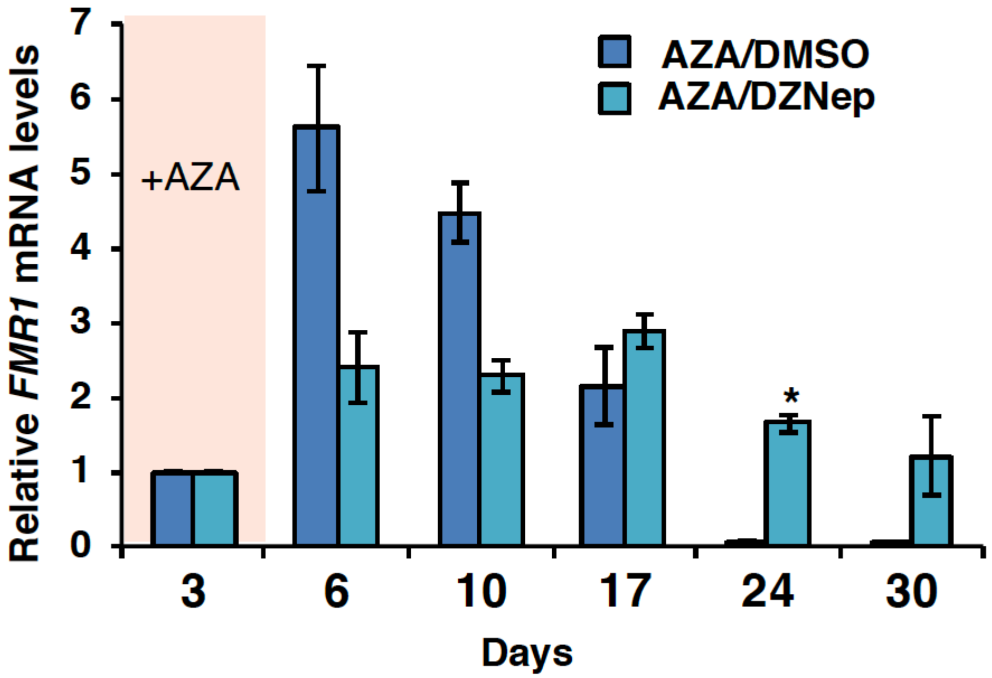

Figure S3. Effect of DZNep treatment in delaying re-silencing in FXS cell line GM0032B. Cells were treated with 10 μM AZA for 3 days, split into two and treated with either DMSO or 5 μM DZNep, added fresh every 3 d for 30 d. The *FMR1* mRNA levels were first normalized to the levels of *GUS* mRNA and are shown here relative to levels at day 3. Data shown are from two independent treatments. Statistical significance was calculated using student's *t* test (paired) and $p < 0.05$ are indicated by an asterix.

1 2 3 4 5 6
cgccactgagtgcacctctgcagaaatgggcgttctggccctcgcgaggcagtgcgacctgtcaccgcccttcagccttcccgccctccacca
7 8 9 10 11 12 13 14 15 16 17 18 19 20 21 22
agcccgcgcacgcccggcccgcgcgtctgtctttcgacccggcaccccggccggttcccagcagcgcgcatgcgcgcgctcccaggccac
ttgaagagagagggcgggccgaggggctgagcccgcgggggagggaacagcgttgatcacgtgacgtggtttcagtgtttac

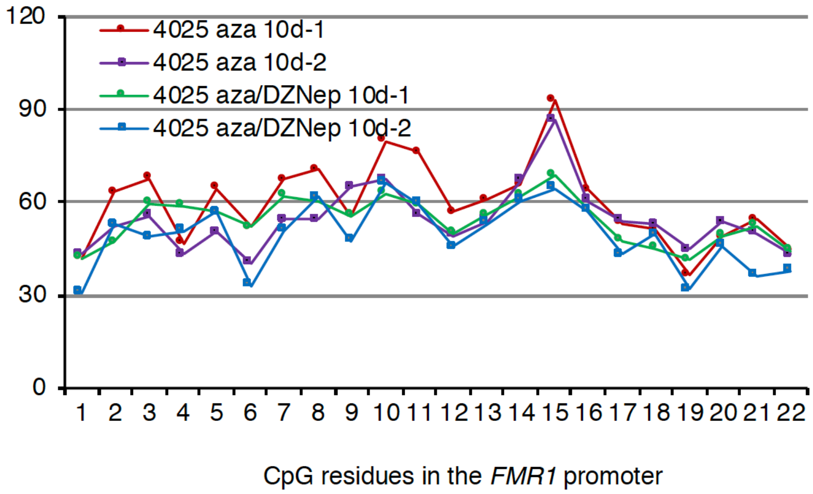

Figure S4. Effect of DZNep treatment on DNA methylation levels in GM04025 cells. Genomic DNA from AZA and AZA/DZNep treated cells was isolated at 10 days and the DNA methylation status was determined by pyrosequencing. Results are shown for two replicates for each treatment. The top part of the panel shows the sequence of the region assayed indicating the 22 CpG residues interrogated in this assay. The red text refers to the conserved transcription factor binding sites in the *FMR1* promoter.

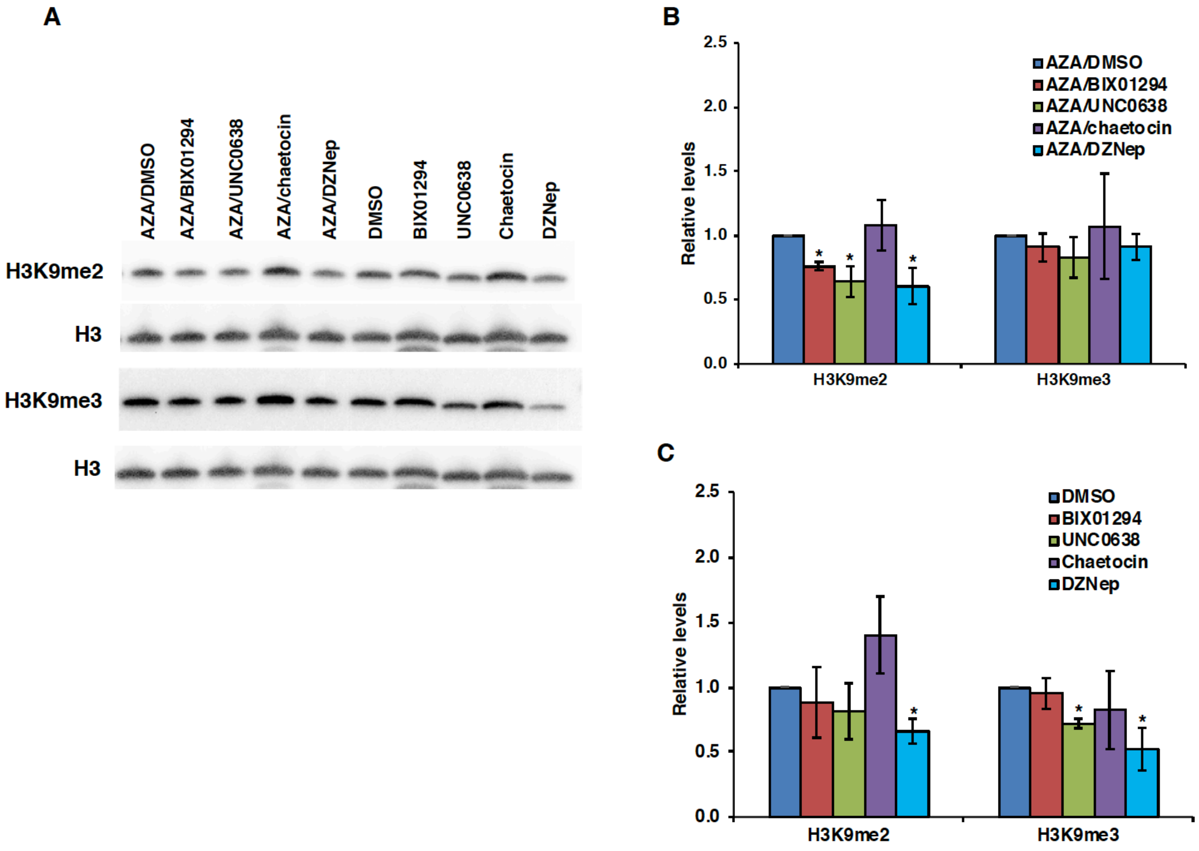

Figure S5. Western blot analyses for H3K9me2 and H3K9me3 in FXS cell line GM0032B. **(A)** A representative western blot. **(B,C)** Quantitation from three independent western blot experiments. Cells were treated with either HMT inhibitors alone or in combination with AZA as described under Materials and Methods. Total cell lysates were prepared after 48 h treatment with HMT inhibitors alone or at day 5 after AZA/HMT inhibitor treatment. Total H3 levels were used for normalization. Error bars represent standard deviation and statistically significant differences are indicated by an asterix ($p < 0.05$).

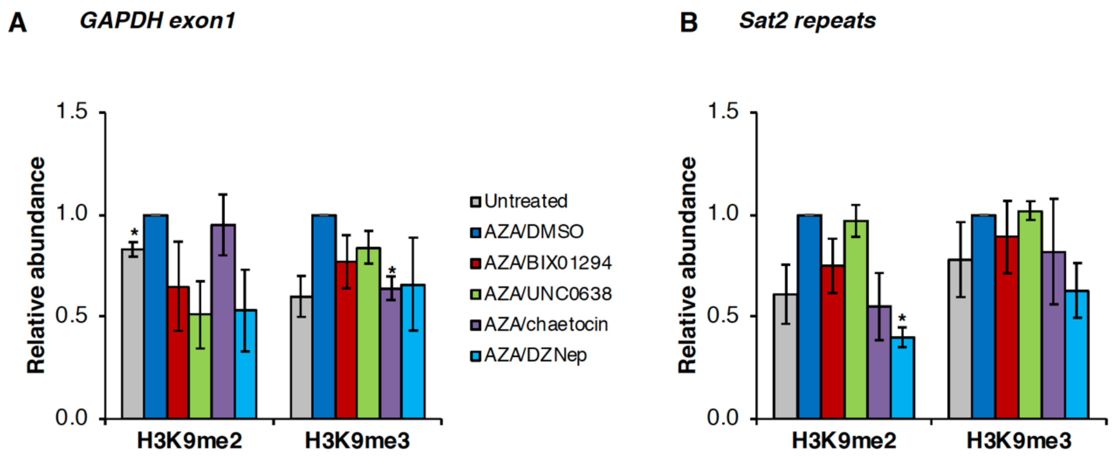

Figure S6. (A,B) Effect of HMT inhibitors on the levels of H3K9me2 and H3K9me3 on the *GAPDH exon1* **(A)** and *Sat2 repeats* **(B)** in the FXS cell line, GM04025. Cells were treated with 10 μM AZA for 72 h followed by indicated HMT inhibitors as described in the legend for Figure 3. Chromatin was prepared at day 5 from treated and untreated cells and used in ChIP assay. Abundance of H3K9me2 and H3K9me3 in AZA/HMT inhibitor treated cells is shown relative to AZA/DMSO treated cells. Data shown is an average of three independent experiments and error bars represent standard error of the mean (SEM). Statistically significant differences are indicated by an asterix ($p < 0.05$).
